# The role of emotion regulation in the relationship between mindfulness and risk factors for disordered eating: A longitudinal mediation analysis

**DOI:** 10.1002/eat.23849

**Published:** 2022-11-11

**Authors:** Emma L. Osborne, Ben Ainsworth, Paul Chadwick, Melissa J. Atkinson

**Affiliations:** ^1^ Department of Psychology University of Bath Bath UK; ^2^ School of Psychology University of Southampton Southampton UK

**Keywords:** body image, eating disorders, emotion regulation, longitudinal design, mechanisms, mediation analysis, mindfulness, mindfulness‐based intervention, negative affect, weight and shape concerns

## Abstract

**Objective:**

Evidence suggests mindfulness may reduce risk factors for disordered eating. However, mechanisms of change in this relationship are unclear. This longitudinal study tested whether emotion regulation mediates the prospective associations between mindfulness and two proximal risk factors for disordered eating: weight and shape concerns, and negative affect.

**Method:**

This study is a secondary analysis of data collected within an eating disorder prevention trial. Adolescent girls (*N* = 374, *M*_age_  = 15.70, *SD* = 0.77) completed self‐report measures of mindfulness, emotion regulation, weight and shape concerns, and negative affect at baseline, 2 months following baseline, and 7 months following baseline. Path analyses were computed to test hypothesized indirect effects using confidence intervals based on 5000 bootstrap samples.

**Results:**

Higher baseline mindfulness predicted lower weight and shape concerns and negative affect at 7 months via a mediator of better emotion regulation at 2 months. This effect remained while controlling for earlier measurements of the mediator and outcome in the model of negative affect but not weight and shape concerns.

**Discussion:**

Emotion regulation may be an important mechanism explaining how mindfulness influences negative affect. Efforts should be made to intervene on mindfulness and emotion regulation in prevention and early intervention programmes for eating disorders and other psychiatric conditions.

**Public Significance:**

Research has shown that mindfulness can help to reduce some of the risk of developing an eating disorder. This study explored whether mindfulness reduces some of this risk by helping people to better manage their emotions. Understanding this process can help us to develop better mindfulness‐based strategies to support people who are at risk of developing an eating disorder.

## 1 INTRODUCTION

Mindfulness is increasingly investigated within eating disorder prevention and early intervention (Beccia et al., 2018). Examining processes through which mindfulness affects risk can support more efficient and effective prevention of eating disorders and other psychiatric conditions (Ahuvia et al., 2022). Emotion regulation is commonly proposed to be a mechanism of mindfulness (e.g., Chiesa et al., 2014; Hölzel et al., 2011; Roemer et al., 2015; Vanzhula & Levinson, 2020); however, few longitudinal studies have examined emotion regulation as a mediator. Regarding eating disorders, emotion regulation difficulties are central to psychopathology (Mallorquí‐bagué et al., 2018), frequently identified across theoretical models (Pennesi & Wade, 2016), and linked to risk (Sim & Zeman, 2006). Two factors provide worthwhile risk targets: weight and shape concerns (i.e., body dissatisfaction, preoccupation, overevaluation in self‐judgment) and negative affect (Jacobi & Fittig, 2010; Pennesi & Wade, 2016). Weight and shape concerns are also a core symptom of eating disorders (DuBois et al., 2017), and negative affect is a non‐specific predictor of psychopathology (Lynch et al., 2021).

Mindfulness may promote more adaptive emotion regulation in different ways. One is that bringing full awareness to current experiences, including those related to the body and appearance, may help individuals detect the need to apply or adjust emotion regulation strategies (Teper et al., 2013). Second, by emphasizing flexible attention, mindfulness may facilitate the use of situation‐appropriate emotion regulation strategies in response to changes in phasic affect (Roemer et al., 2015), such as appearance anxiety and body shame. Third, non‐judgemental qualities of mindfulness may reduce experiential avoidance (Chambers et al., 2009), and in turn mitigate the impact of risk factors in the vulnerability to eating disorder psychopathology (Litwin et al., 2017; Mendes et al., 2017).

We used a longitudinal design to test whether prospective associations between mindfulness and weight and shape concerns and negative affect are mediated by emotion regulation. We examined these relationships in female adolescents who participated in a prevention study. Eating disorders typically develop during adolescence (Nagl et al., 2016), making this a critical period to focus prevention and early intervention efforts. We hypothesized that higher mindfulness at Time 1 would predict better emotion regulation at Time 2, in turn leading to lower weight and shape concerns and negative affect at Time 3.

## 2 METHOD

### 2.1 Procedure

We conducted a secondary analysis of data from an eating disorder prevention trial, described in Atkinson and Wade (2015). Briefly, the data are from a school‐based randomized controlled study that evaluated a 3‐week mindfulness‐based intervention, compared to a dissonance‐based intervention and classes‐as‐usual control, for reducing eating disorder risk factors. Australian girls' schools were invited to take part via email and follow‐up telephone contact, and students from participating schools were invited via letters to parents and students requesting opt‐in informed consent and assent, respectively. There were no exclusion criteria.

In the present study, we used data from all participants by collapsing groups because there were no differences between groups on any variables in the mediation models, nor any interactions between group and time (Supporting Information—Appendix S1). Group allocation did not influence levels of mindfulness, emotion regulation, weight and shape concerns, or negative affect, over time for the whole sample. The design and analysis plan were pre‐registered prior to analysis (https://osf.io/2npby). All procedures received institutional ethical approval (Ref: 21–153) and approval from the Catholic Education Office and school principals.

### 2.2 Participants

Participants were 374 girls aged 14–18 (*M*_age_ = 15.70, *SD* = 0.77; *M*_BMI_ = 20.76, *SD* = 2.91). Most participants were Caucasian (82.9%), while the remaining participants identified as Asian (7.5%), African (0.5%), or Other (3.7%).

### 2.3 Measures

Participants self‐reported their age, height, weight, ethnicity, and completed outcome measures at baseline, 2 months following baseline, and 7 months following baseline. Further details regarding measures, including example items, response options, and psychometric properties, are provided in Supporting Information—Appendix S1. Table 1 provides internal consistencies.

**TABLE 1:** Descriptive statistics and bivariate correlations

Variable	*M* (*SD*)	*α*	1	2	3	4	5	6	7	8	9
1. T1 Mindfulness	3.63 (0.80)	0.89	–								
2. T1 Emotion regulation difficulties	2.45 (0.72)	0.91	−0.75	–							
3. T2 Emotion regulation difficulties	2.28 (0.70)	0.93	−0.57	0.69	–						
4. T1 Weight and shape concerns	2.69 (1.68)	0.95	−0.42	0.45	0.43	–					
5. T2 Weight and shape concerns	2.23 (1.71)	0.96	−0.34	0.41	0.52	0.78	–				
6. T3 Weight and shape concerns	2.40 (1.75)	0.97	−0.35	0.38	0.48	0.77	0.85	–			
7. T1 Negative affect	2.17 (0.85)	0.95	−0.67	0.70	0.56	0.49	0.46	0.43	–		
8. T2 Negative affect	2.12 (0.92)	0.97	−0.54	0.58	0.73	0.45	0.56	0.53	0.69	–	
9. T3 Negative affect	2.24 (0.91)	0.96	−0.55	0.59	0.68	0.46	0.53	0.59	0.61	0.73	–

*Note*: All correlations *p* < .001. T1 = baseline, T2 = 2 months following baseline, T3 = 7 months following baseline.

#### 2.3.1 Mindfulness

Child and Adolescent Mindfulness Measure (Greco et al., 2011; 10 items, higher scores indicate greater mindfulness).

#### 2.3.2 Emotion regulation

Difficulties in Emotion Regulation Scale Short Form (Kaufman et al., 2016; 18 items, higher scores indicate greater difficulties in emotion regulation).

#### 2.3.3 Weight and shape concerns

Eating Disorder Examination Questionnaire (Fairburn & Beglin, 1994) Weight/Shape Concerns scale (12 items, higher scores indicate greater concerns).

#### 2.3.4 Negative affect

Positive and Negative Affect Schedule‐Expanded (Watson & Clark, 1994) Sadness, Guilt, and Fear/Anxiety subscales (17 items, higher scores indicate greater negative affect).

### 2.4 Data analyses

Data were screened for coding errors and missing values. Coded values were within the appropriate range and missingness ranged from 8.8 to 21.9% for variables across models. The probability of the patterns of missing values diverging from randomness was >0.05, thus data missing completely at random was inferred. We used the expectation‐maximization algorithm to impute missing values. We ran bivariate correlations among all variables and performed mediation analyses using the PROCESS macro for SPSS (Hayes, 2022). We entered baseline mindfulness as the predictor, 2‐month emotion regulation as the mediator, and 7‐month weight and shape concerns and negative affect as outcomes in separate models. Repeated analyses controlled for baseline emotion regulation, and baseline and 2‐month weight and shape concerns and negative affect. We used bootstrapping for 95% confidence intervals (CIs) and standard errors of indirect effects from 5000 samples. More information about data preparation and analysis is provided in Supporting Information—Appendix S1.

## 3 RESULTS

### 3.1 Descriptive and correlation analyses

Table 1 displays descriptive statistics and bivariate correlations. All variables were significantly correlated (absolute value coefficients between 0.34 and 0.85).

### 3.2 Mediation analyses

**FIGURE 1:**
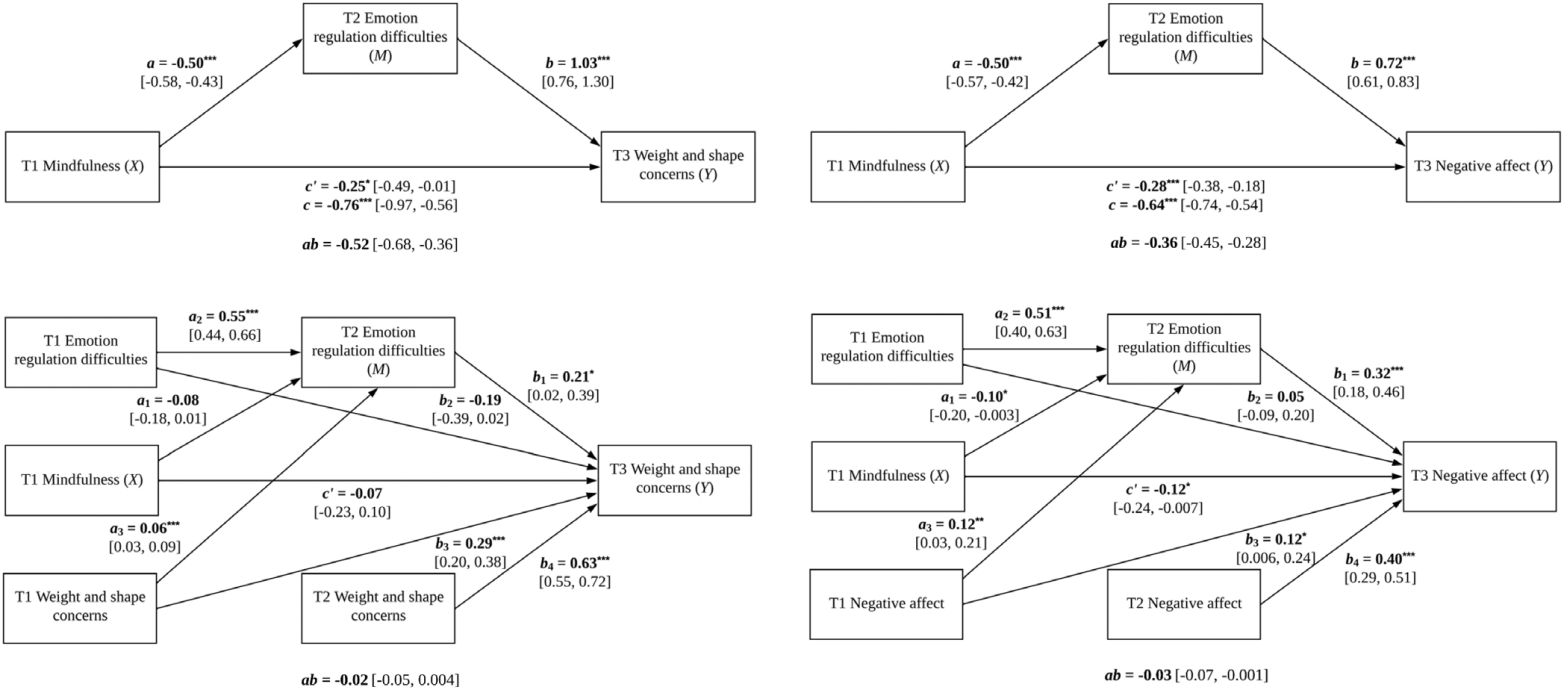
Longitudinal mediation models. Coefficients are unstandardized. ***p < .001. **p < .01. *p < .05. T1 = baseline, T2 = 2 months following baseline, T3 = 7 months following baseline. Values in square brackets are 95% CIs. *c* = X–Y total effect, *c’* = X–Y direct effect, *ab* = X–M–Y indirect effect (bootstrapped 95% CIs not containing zero indicate a significant indirect effect).

Figure 1 presents the longitudinal mediation models of weight and shape concerns (left) and negative affect (right). As shown in the top panel, path estimates indicated significant inverse relationships between baseline mindfulness and 7‐month weight and shape concerns and negative affect. Girls reporting higher baseline mindfulness experienced lower weight and shape concerns and negative affect at 7 months. Additionally, girls with higher baseline mindfulness experienced better emotion regulation at 2 months, and girls with better emotion regulation at 2 months reported lower weight and shape concerns and negative affect at 7 months. CIs for indirect effects were entirely below zero, indicating that emotion regulation at 2 months mediated associations between baseline mindfulness and 7‐month weight and shape concerns and negative affect. Direct effects were significant: after accounting for indirect effects through emotion regulation at 2 months, higher baseline mindfulness was associated with lower weight and shape concerns and negative affect at 7 months. Together, baseline mindfulness and 2‐month emotion regulation explained a significant proportion of variance in 7‐month weight and shape concerns and negative affect.

As shown in the bottom left portion of Figure 1, baseline mindfulness was unrelated to 2‐month emotion regulation after controlling for baseline emotion regulation and weight and shape concerns. However, 2‐month emotion regulation was significantly associated with 7‐month weight and shape concerns after controlling for baseline emotion regulation and baseline and 2‐month weight and shape concerns. Girls reporting higher baseline mindfulness did not experience improvements in emotion regulation at 2 months; however, girls with better emotion regulation at 2 months reported reductions in weight and shape concerns at 7 months. A CI for the indirect effect contained zero, indicating that residualized emotion regulation at 2 months did not mediate the association between baseline mindfulness and residualized weight and shape concerns at 7 months. The direct effect was nonsignificant: after controlling for previous levels of emotion regulation and weight and shape concerns, baseline mindfulness was not associated with weight and shape concerns at 7 months. Previous levels of emotion regulation and weight and shape concerns explained a significant proportion of variance in weight and shape concerns at 7 months.

As shown in the bottom right portion of Figure 1, baseline mindfulness was significantly associated with 2‐month emotion regulation after controlling for baseline emotion regulation and negative affect, and 2‐month emotion regulation was significantly associated with 7‐month negative affect after controlling for baseline emotion regulation and baseline and 2‐month negative affect. Girls reporting higher baseline mindfulness experienced improvements in emotion regulation at 2 months, and in turn, girls with better emotion regulation at 2 months reported reductions in negative affect at 7 months. A CI for the indirect effect was entirely below zero, indicating that residualized emotion regulation at 2 months mediated the association between baseline mindfulness and residualized negative affect at 7 months. The direct effect was significant: after accounting for the indirect effect through emotion regulation at 2 months, higher baseline mindfulness was associated with lower negative affect at 7 months. Previous levels of emotion regulation and negative affect explained a significant proportion of variance in negative affect at 7 months.

Further details, including standard errors and exact *p* values, are provided in Supporting Information—Appendix S1. Here, we also report a sensitivity analysis using a different method to manage missing data, with interpretation of our findings remaining consistent.

## 4 DISCUSSION

Several key findings emerged from the present study. First, emotion regulation mediated the prospective association between mindfulness and weight and shape concerns. Specifically, higher mindfulness predicted lower weight and shape concerns via better emotion regulation—the first longitudinal data to offer support for this relationship. The effect subsided after controlling for previous emotion regulation and weight and shape concerns, perhaps because this analysis left too little unexplained variance for the predictor variables to explain (Jose, 2016). Alternatively, since body dissatisfaction seems relatively stable from mid‐adolescence onward (Wang et al., 2019), a longer observation period may be required to capture sufficient change in outcomes to test for an indirect effect. Nonetheless, better emotion regulation was temporally related to lower weight and shape concerns while controlling for earlier measurements, which indicates that intervening on emotion regulation via other methods may help to reduce weight and shape concerns.

Second, emotion regulation mediated the prospective association between mindfulness and negative affect, even after controlling for previous levels of emotion regulation and negative affect. Individuals with increased awareness and non‐judgemental acceptance of ongoing thoughts and feelings were less likely to experience subsequent emotion regulation difficulties and, in turn, negative affect. This is consistent with theoretical frameworks proposing emotion regulation as a pathway for the psychological effects of mindfulness (e.g., Chiesa et al., 2014; Hölzel et al., 2011; Roemer et al., 2015; Vanzhula & Levinson, 2020). It is also consistent with evidence that mindfulness increases positive affect through emotion regulation in patients with social anxiety disorder (Garland et al., 2017). Our results extend this evidence to negative affect in a non‐clinical population.

The disparity in findings across our two outcomes may also indicate that unique processes underlie the influence of mindfulness on constructs related to negative affect, or on non‐specific risk factors more generally, compared to those related to weight and shape concerns. Other factors occupying central positions in models of disordered eating, such as sociocultural pressures and thin‐ideal internalization in the dual‐pathway model (Stice, 2001), may have stronger mediating roles in the effect of mindfulness on eating disorder‐specific risk factors, such as weight and shape concerns. Alternatively, emotion regulation may mediate the influence of mindfulness on factors that come later in the developmental trajectory, in this case, negative affect, dieting, and disordered eating behaviors.

This study implemented a robust test of mediation using a three‐wave autoregressive model with bootstrapping. However, participants were predominantly Caucasian, and two‐thirds took part in an eating disorder prevention programme. Our findings may not generalize to other groups or outside of a prevention context. Racial and ethnic diversity is an important goal for future eating disorder research given that etiology, presentation, and treatment may vary across racial and ethnic groups (e.g., Goel et al., 2020) whilst current research often recruits predominantly White samples (Egbert et al., 2022). Additionally, while our longitudinal analyses provide support for the directionality of relationships predicted by theory, it is possible that associations exist in another order. Future research may benefit from examining all possible orderings of variables over time.

These findings indicate that mindfulness‐based intervention focused on improving emotion regulation may be particularly effective at reducing negative affect, although further work should verify benefits for weight and shape concerns. Future research should investigate how to effectively target emotion regulation and evaluate the potential benefits of doing so.

## AUTHOR CONTRIBUTIONS

**Emma L. Osborne:** Conceptualization; formal analysis; funding acquisition; methodology; writing – original draft; writing – review and editing. **Ben Ainsworth:** Methodology; supervision; writing – review and editing. **Paul Chadwick:** Conceptualization; methodology; supervision; writing – review and editing. **Melissa J. Atkinson:** Conceptualization; funding acquisition; methodology; supervision; writing – review and editing.

## FUNDING INFORMATION

This work was supported by the Economic and Social Research Council South West Doctoral Training Partnership [grant number ES/P000630/1].

## CONFLICT OF INTEREST

The authors declare no competing interests.

## Supporting information


**Appendix S1.** Supporting Information

## Data Availability

The data are available from the corresponding author upon reasonable request.
