# Supplementary material for: The role of emotion regulation in the relationship between mindfulness and risk factors for disordered eating: A longitudinal mediation analysis
Source: Int J Eat Disord. 2022 Nov 11;56(2):458–63. doi: 10.1002/eat.23849 (PMC10099542; doi:10.1002/eat.23849)
Supplement: Supplementary file 1 — Appendix S1. Supporting Information [file EAT-56-458-s001.docx]

**Article title**: The role of emotion regulation in the relationship between mindfulness and risk factors for disordered eating: A longitudinal mediation analysis

**Journal name**: *International Journal of Eating Disorders (IJED)*

**Author names**: Emma L. Osborne, Ben Ainsworth, Paul Chadwick, Melissa J. Atkinson

**Corresponding author**: Emma L. Osborne, Department of Psychology, University of Bath, Claverton Down, Bath, BA2 7AY, UK; Email: elo25@bath.ac.uk

**Contents**

[1 Supporting Information 2](#_Toc117704094)

[1.1 Group Allocation 2](#_Toc117704095)

[1.2 Measures 2](#_Toc117704096)

[1.2.1 Mindfulness 2](#_Toc117704097)

[1.2.2 Emotion Regulation 3](#_Toc117704098)

[1.2.3 Weight and Shape Concerns 3](#_Toc117704099)

[1.2.4 Negative Affect 4](#_Toc117704100)

[1.3 Data Preparation 4](#_Toc117704101)

[1.4 Data Analysis 7](#_Toc117704102)

[1.5 Results 7](#_Toc117704103)

[1.6 Sensitivity Analyses 8](#_Toc117704104)

[1.6.1 Scenario 1 8](#_Toc117704105)

[1.6.2 Scenario 2 11](#_Toc117704106)

[2 Figures 14](#_Toc117704107)

[3 Tables 17](#_Toc117704108)

[4 References 26](#_Toc117704109)

[5 Appendix A 30](#_Toc117704110)

[6 Appendix B 37](#_Toc117704111)

# Supporting Information

## Group Allocation

We performed one-way analyses of variances (ANOVAs) to examine whether there were any differences between groups (acceptance, dissonance, control) on any variables in the mediation models (mindfulness, emotion regulation, weight and shape concerns, negative affect) across time points (baseline, 2 months after baseline, 7 months after baseline). There were no differences between groups on any of the variables in the mediation models across time (see **Table S1**). We also performed 3 (group) × 3 (time) mixed ANOVAs to determine whether any changes in the variables in the mediation models were a result of an interaction between group and time. There was no interaction between group and time on any of the variables in the mediation models: mindfulness, *F*(3.84, 711.37) = 1.62, *p* = .169, η_p_^2^ = .009; emotion regulation, *F*(3.78, 700.84) = 0.88, *p* = .472, η_p_^2^ = .005; weight and shape concerns, *F*(3.82, 709.37) = 0.81, *p* = .513, η_p_^2^ = .004; negative affect, *F*(3.89, 722.05) = 0.66, *p* = .619, η_p_^2^ = .004 (**Figure S1**). Group allocation did not influence levels of mindfulness, emotion regulation, weight and shape concerns, or negative affect, over time for the whole sample.

## Measures

### Mindfulness

Mindfulness was assessed using the Child and Adolescent Mindfulness Measure (CAMM; Greco et al., 2011), specifically developed for and validated in school-aged youth. The CAMM has 10 items, and these were adapted from mindfulness constructs also captured in the FFMQ (observe, acting with awareness, non-judging). Each item (e.g., “I push away thoughts that I don’t like”) was assessed generally and scored from 1 (*never true*) to 5 (*always true*). Total mindfulness was calculated by taking the mean (after all items were reverse scored), with higher scores indicating greater mindfulness. The CAMM has shown adequate internal consistency and scores correlate negatively with adverse outcomes (Greco et al., 2011). In the current sample, internal consistency was .89 at baseline and .92 at 2 months and 7 months.

### Emotion Regulation

Emotion regulation was assessed using the Difficulties in Emotion Regulation Scale–Short Form (DERS‑SF; Kaufman et al., 2016). The DERS-SF has six subscales (strategies, non-acceptance, impulse, goals, awareness, clarity) with three items each. Each item (e.g., “I am confused about how I feel”) was assessed generally and scored from 1 (*almost never*) to 5 (*almost always*). Total emotion regulation was calculated by taking the mean of 18 items (three items were reverse scored), with higher scores indicating greater difficulties. The DERS-SF has shown good reliability and concurrent validity in both adult and adolescent samples (Kaufman et al., 2016). In the current sample, internal consistency was .91 at baseline and .93 at 2 months and 7 months.

### Weight and Shape Concerns

Weight and shape concerns were assessed using two relevant subscales from the Eating Disorder Examination–Questionnaire (EDE‑Q; Fairburn & Beglin, 1994). The Weight Concern and Shape Concern subscales have five and eight items, respectively. Each item (e.g., “How dissatisfied have you been with your weight?”) was assessed over the previous 28 days and scored from 0 (*not at all*) to 6 (*markedly*). Total weight and shape concerns was calculated by taking the mean of 12 items (one shared item), with higher scores indicating greater concerns. The EDE-Q is a widely accepted measure of eating disorder psychopathology and has shown high internal consistency and convergence with the interview-based EDE (Berg et al., 2012). It is worth noting that the four-factor structure of the EDE-Q has been subject to criticism (e.g., Rand-Giovannetti et al., 2020), although the focus of this article was on the Weight Concern and Shape Concern subscales only. Items from these two subscales have consistently been found to load onto a single factor (Berg et al., 2012). The 12 unique items from these two subscales are commonly averaged to create a composite measure of Weight/Shape Concerns (e.g., Mond et al., 2007; Trompeter et al., 2022; Van Zutven et al., 2015; Wilksch & Wade, 2009), and this composite has demonstrated good internal consistency among adolescents (Gall et al., 2016; Mond et al., 2014). In the current sample, internal consistency was .95 at baseline, .96 at 2 months, and .97 at 7 months.

### Negative Affect

Negative affect was assessed using the sadness, guilt, and fear/anxiety subscales of the Positive and Negative Affect Schedule–Expanded (PANAS-X; Watson & Clark, 1994). These subscales have five, six, and six items, respectively. Each item (e.g., ashamed) was assessed based on the extent felt during the past week and scored from 1 (*very slightly or not at all*) to 5 (*extremely*). Total negative affect was calculated by taking the mean of 17 items, with higher scores indicating greater negative affect. The PANAS-X has shown high internal consistency and predictive validity for bulimic symptom onset (Stice & Agras, 1998). In the current sample, internal consistency was .95 at baseline, .97 at 2 months, and .96 at 7 months.

## Data Preparation

Our pre-registered design and analysis plan on the Open Science Framework (OSF: <https://osf.io/2npby>) provides further justification for our data cleaning methods. Prior to running the primary analyses, we screened data for coding errors and missing values. All coded values were within the appropriate range. We excluded all participants who had not completed any of the measures for the study (*n* = 2) giving us a final sample of 374.

In the model of weight and shape concerns using earlier measurements (aka “lags”) as covariates, there were 215 complete cases and 159 cases with incomplete data. Rates of missingness ranged from 16.7 to 83.3% (mean of 30.9%) for cases and from 8.8 to 19.3% (mean of 13.1%) for variables. Further inspection of the data suggested that participants with greater weight and shape concerns at baseline and 7 months were less likely to report on emotion regulation and weight and shape concerns at 2 months. However, the probability of the pattern of missing values diverging from randomness was greater than .05 (MCAR χ² = 102.72, *df* = 86, *p* = .106), thus data missing completely at random (MCAR) was inferred.

In the model of negative affect using earlier measurements (aka “lags”) as covariates, there were 205 complete cases and 169 cases with incomplete data. Rates of missingness ranged from 16.7 to 83.3% (mean of 31.6%) for cases and from 9.1 to 21.9% (mean of 14.3%) for variables. Further inspection of the data suggested that participants with higher negative affect at 7 months were less likely to report on emotion regulation at 2 months. However, the probability of the pattern of missing values diverging from randomness was greater than .05 (MCAR χ² = 91.40, *df* = 86, *p* = .325), thus MCAR was inferred.

We used the expectation-maximisation (EM) algorithm (100 iterations) to impute missing values in both longitudinal mediation models because the EM algorithm can produce reliable parameter estimates (i.e., within 1% of the original value) at up to 50% MCAR (Scheffer, 2002), indicating it can produce accurate results even at this high percentage of missingness when the missing data are ignorable (i.e., MCAR or missing at random [MAR]).

Examination of boxplots, histograms, and normal probability plots indicated approximately normal distributions for all variables in the mediation models across all time points. Absolute values of skewness and kurtosis did not exceed the recommended critical values for sample sizes greater than 300 (i.e., were not larger than 2 and 7, respectively; see **Table S2**), indicating variables did not substantially depart from normality (Kim, 2013).

We detected univariate outliers using the median absolute deviation (MAD), which is a robust dispersion measure that is highly insensitive to the presence of outlying values (Leys et al., 2013). Consistent with Leys et al.'s (2013) recommendation, we used the median ± 2.5*MAD. We identified 12 participants as outliers in terms of mindfulness at baseline; 13 in terms of emotion regulation at baseline and 10 in terms of emotion regulation at 2 months; none in terms of weight and shape concerns across time; 14 in terms of negative affect at both baseline and 2 months, and 6 in terms of negative affect at 7 months (see **Table S3**). We winsorized outliers by converting them to the highest or lowest value not considered an outlier. Winsorization preserves information that the outlier had among the highest or lowest values in a distribution while protecting against some of their harmful effects (e.g., exerting a disproportionate influence on correlations; Reifman & Keyton, 2012).

We screened for multivariate outliers by computing Mahalanobis distances from multiple regression analyses and evaluating them as χ^2^ with probability criterion *p* < .001 and degrees of freedom equal to the number of variables (Tabachnick & Fidell, 2019). In the models of weight and shape concerns, we identified no multivariate outliers in the model without covariates and two multivariate outliers in the model using earlier measurements (aka “lags”) as covariates (*df* = 3 and 6, respectively). In the models of negative affect, we identified one multivariate outlier in the model without covariates and five multivariate outliers in the model using earlier measurements (aka “lags”) as covariates (*df* = 3 and 6, respectively). Since multivariate outliers may distort results of statistical analyses in almost any direction (Tabachnick & Fidell, 2019), we filtered them from the corresponding analyses.

We tested data for linearity, independent errors, homoscedasticity, and multicollinearity (see **Table S4**). Examination of scatter plots indicated approximately linear associations between variables. Durbin–Watson statistics for variables in each model were close to 2 and between 1–3, indicating independent errors (Field, 2013). Scatterplots of residuals terms indicated that the distribution of data points was approximately equal at each level of the predicted value, suggesting data were homoscedastic. Variance inflation factors were < 10 (Myers, 1990), tolerance statistics were > 0.2 (Menard, 1995), and correlation coefficients (absolute values) for bivariate correlations between predictor variables (i.e., mindfulness and emotion regulation) did not exceed .75 (i.e., no substantial correlations of |*r*| > .9 between predictors), indicating that they were not perfectly collinear (Field, 2013).

## Data Analysis

We ran bivariate correlations among all study variables. Mediation analyses were performed using a regression-based bootstrap approach using the PROCESS macro for SPSS (Hayes, 2022). We used 20220905 as the random seed for all mediation analyses to ensure results are reproducible. We did not include BMI or age as covariates because they were not related to both the predictors and the outcomes (see **Table S5**), which means adjusting for them would have no effect other than using degrees of freedom (IJED, 2019). We used bootstrapping for 95% confidence intervals (CIs) and standard errors (*SE*s) of indirect effects from 5000 samples, with CIs not including 0 considered significant. Bootstrapping provides more accurate estimates and greater power than traditional methods (Mackinnon et al., 2004).

## Results

In the model of weight and shape concerns, a 95% CI for the indirect effect was entirely below zero, *ab* = -0.52 [-0.68, -0.36], *SE* = 0.08. The effect did subside after controlling for previous levels of emotion regulation and weight and shape concerns: *ab* = -0.02 [-0.05, 0.004], *SE* = 0.01. In the model of negative affect, a 95% CI for the indirect effect was entirely below zero, *ab* = -0.36 [-0.45, -0.28], *SE* = 0.04. The effect remained significant after controlling for previous levels of emotion regulation and negative affect: *ab* = -0.03 [-0.07, -0.001], *SE* = 0.02. **Table S6** and **Table S7** provide *SE*s and exact *p* values for the models of weight and shape concerns and negative affect, respectively.

## Sensitivity Analyses

We performed two sensitivity analyses to explore the result of the mediation analyses under alternative scenarios for handling missing data.

### Scenario 1

First, we used listwise deletion, also known as complete case analysis, to manage all missing data. For each mediation model, we included only the cases for which there were no missing values on any of the variables in that model. We performed all data preparation and mediation analyses separately for each model to make use of all available data. There were 233 participants in the complete case sensitivity analyses (*M*_age_ = 15.66, *SD* = 0.75; *M*_BMI_ = 20.68, *SD* = 2.80): for weight and shape concerns, there were 230 participants in the model without covariates and 213 participants in the model using earlier measurements (aka “lags”) as covariates; for negative affect, there were 225 participants in the model without covariates and 204 participants in the model using earlier measurements (aka “lags”) as covariates.

We handled univariate and multivariate outliers in the same way as in the main analysis (i.e., using the median absolute deviation [MAD] and winsorization for univariate outliers, and Mahalanobis distances and exclusion for multivariate outliers; see **1.3** for details about these methods). We used Python to automate univariate outlier detection and treatment. We used pandas to load the dataset and apply winsorization, and SciPy to calculate the MAD (see **Appendix A** for detailed findings from each model). In the models of weight and shape concerns, we identified no multivariate outliers in the model without covariates and two multivariate outliers in the model using earlier measurements (aka “lags”) as covariates (*df* = 3 and 6, respectively). In the models of negative affect, we identified no multivariate outliers in the model without covariates and one multivariate outlier in the model using earlier measurements (aka “lags”) as covariates (*df* = 3 and 6, respectively).

**3.4.1.1 Results**. Using listwise deletion to manage all missing data did not change the interpretation of our findings (detailed results below).

***Weight and Shape Concerns.*** The mediation models are displayed in **Figure S2** (see **Table S8** for *SE*s and exact *p* values). In the first model, path estimates indicated a significant inverse relationship between baseline mindfulness and 7-month weight and shape concerns, such that girls reporting higher baseline mindfulness experienced lower levels of weight and shape concerns at 7 months. Additionally, girls with higher baseline mindfulness experienced fewer emotion regulation difficulties at 2 months, and girls experiencing lower emotion regulation difficulties at 2 months reported lower levels of weight and shape concerns at 7 months. A 95% CI for the indirect effect was entirely below zero, *ab* = -0.56 [-0.78, -0.34], *SE* = 0.11, indicating that emotion regulation at 2 months mediated the association between baseline mindfulness and 7-month weight and shape concerns. The direct effect was nonsignificant, such that after accounting for the indirect effect through emotion regulation at 2 months, baseline mindfulness did not predict 7-month weight and shape concerns. Baseline mindfulness and 2-month emotion regulation together explained a significant proportion (25.7%) of variance in 7-month weight and shape concerns, *R*^2^ = .257, *F*(2, 227) = 39.31, *p* < .001.

In the second model, baseline mindfulness significantly predicted 2-month emotion regulation after controlling for baseline emotion regulation and weight and shape concerns, and 2-month emotion regulation significantly predicted 7-month weight and shape concerns after controlling for baseline emotion regulation and baseline and 2-month weight and shape concerns. Girls reporting higher baseline mindfulness experienced improvements in emotion regulation at 2 months, and in turn, girls with better emotion regulation at 2 months reported reductions in weight and shape concerns at 7 months. A 95% CI for the indirect effect contained zero, *ab* = -0.06 [-0.14, 0.002], *SE* = 0.04, indicating that residualised emotion regulation at 2 months did not mediate the association between baseline mindfulness and residualised weight and shape concerns at 7 months. The direct effect was nonsignificant, indicating that after controlling for previous levels of emotion regulation and weight and shape concerns, baseline mindfulness was not associated with weight and shape concerns at 7 months. Previous levels of emotion regulation and weight and shape concerns explained a significant proportion (76.2%) of variance in weight and shape concerns at 7 months, *R*^2^ = .762, *F*(5, 207) = 132.48, *p* < .001.

***Negative Affect.*** The mediation models are displayed in **Figure S3** (see **Table S9** for *SE*s and exact *p* values). In the first model, path estimates indicated a significant inverse relationship between baseline mindfulness and 7-month negative affect, such that girls reporting higher baseline mindfulness experienced lower levels of negative affect at 7 months. Additionally, girls reporting higher baseline mindfulness experienced fewer emotion regulation difficulties at 2 months, and girls who experienced fewer emotion regulation difficulties at 2 months reported lower levels of negative affect at 7 months. A 95% CI for the indirect effect was entirely below zero, *ab* = -0.40 [-0.53, -0.28], *SE* = 0.06, indicating that 2-month emotion regulation mediated the association between baseline mindfulness and 7-month negative affect. The direct effect was significant, such that after accounting for the indirect effect through 2-month emotion regulation, higher baseline mindfulness was associated with lower negative affect at 7 months. Together, baseline mindfulness and 2-month emotion regulation explained a significant proportion (52.3%) of variance in 7-month negative affect, *R*^2^ = .523, *F*(2, 222) = 121.49, *p* < .001.

In the second model, baseline mindfulness significantly predicted 2-month emotion regulation after controlling for baseline emotion regulation and negative affect, and 2-month emotion regulation significantly predicted 7-month negative affect after controlling for baseline emotion regulation and baseline and 2-month negative affect. Girls reporting higher baseline mindfulness experienced improvements in emotion regulation at 2 months, and in turn, girls with better emotion regulation at 2 months reported reductions in negative affect at 7 months. A 95% CI for the indirect effect was entirely below zero, *ab* = -0.08 [-0.18, -0.005], *SE* = 0.04, indicating that residualised emotion regulation at 2 months mediated the association between baseline mindfulness and residualised negative affect at 7 months. The direct effect was nonsignificant, such that after accounting for the indirect effect through emotion regulation at 2 months, baseline mindfulness was not associated with negative affect at 7 months. Previous levels of emotion regulation and negative affect explained a significant proportion (60.3%) of variance in negative affect at 7 months, *R*^2^ = .603, *F*(5, 198) = 60.05, *p* < .001.

### Scenario 2

Second, we used listwise deletion to handle whole-wave missingness and the EM algorithm to handle within-wave missingness. Our intention with this sensitivity analysis was to make a trade-off between using all available data and not imputing a high percentage of missing values. In this analysis, we assumed that the mechanism of whole-wave missingness was MCAR because student absence could have been for many, random reasons (e.g., family holiday, medical grounds, changing schools, caring responsibilities) and therefore we considered students who were present at all timepoints to be a simple random sample of the full dataset (i.e., fully representative of the original sample). Under this ignorable missingness mechanism, listwise deletion is unbiased. We also assumed that the mechanism of within-wave missingness was MAR because missing data on individual variables are likely to be related to other measured variables or *observed data* in the mediation models (e.g., non-response due to negative affectivity). Maximum-likelihood single imputation procedures, such as the EM algorithm, provide a suitable way of handling a small percentage of data MAR (Scheffer, 2002). Overall, this approach will allow us to correct as much as possible for potential biases (within the constraints of our chosen software and without knowing the precise missingness mechanism), while also maximising data usability (Cox et al., 2014).

Of the 374 participants who completed at least one of the measures for the study, 262 were present at all three timepoints. There were no differences between participants who were included compared to excluded with respect to demographics or relevant baseline measurements (see **Table S10**), indicating that whole-wave missingness likely reflected random student absences rather than differences that could introduce bias. For these 262 participants, rates of missingness ranged from 11.1 to 44.4% (mean of 16.2%) for cases and from 1.5 to 9.5% (mean of 4.2%) for variables included in the mediation models. The probability of the pattern of missing values diverging from randomness was greater than .05 (MCAR χ² = 158.83, *df* = 162, *p* = .556), thus MCAR was inferred. We used the EM algorithm (100 iterations) to impute the small amount of within-wave missingness.

We handled univariate and multivariate outliers in the same way as in the main analysis (i.e., using the median absolute deviation [MAD] and winsorization for univariate outliers, and Mahalanobis distances and exclusion for multivariate outliers; see **1.3** for details about these methods). We used Python to automate univariate outlier detection and treatment. We used pandas to load the dataset and apply winsorization, and SciPy to calculate the MAD (see **Appendix B** for detailed findings). In the models of weight and shape concerns, we identified no multivariate outliers in the model without covariates and two multivariate outliers in the model using earlier measurements (aka “lags”) as covariates (*df* = 3 and 6, respectively). In the models of negative affect, we identified no multivariate outliers in the model without covariates and one multivariate outlier in the model using earlier measurements (aka “lags”) as covariates (*df* = 3 and 6, respectively).

**3.4.2.1 Results**. Using listwise deletion to handle whole-wave missingness and the EM algorithm to handle within-wave missingness changed the interpretation of one finding: in the model of negative affect using earlier measurements (aka “lags”) as covariates, a 95% CI for the indirect effect contained zero, *ab* = -0.04 [-0.11, 0.02], *SE* = 0.03, indicating that residualised emotion regulation at 2 months did not mediate the association between baseline mindfulness and residualised negative affect at 7 months under this alternative scenario for handling missing data.

# Figures


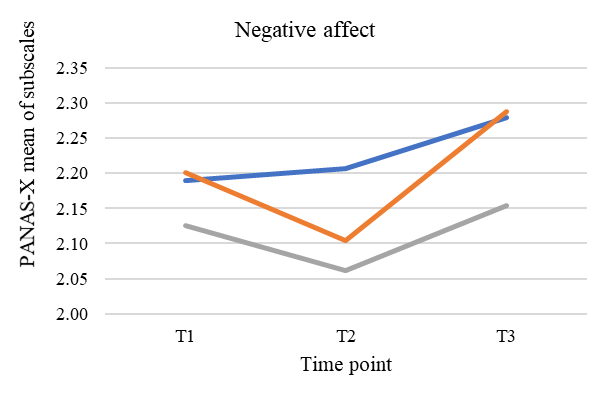

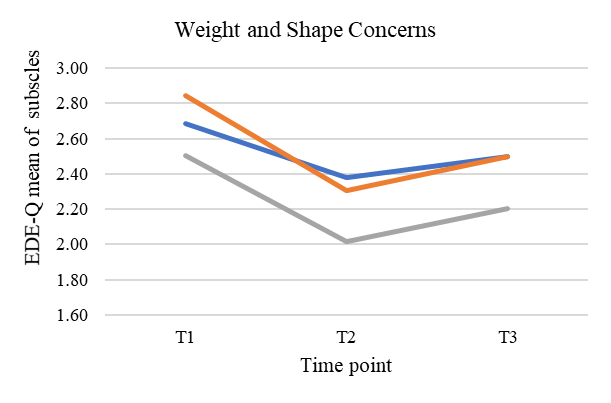

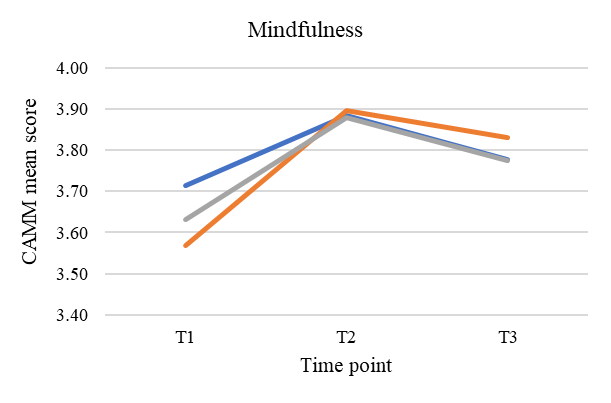

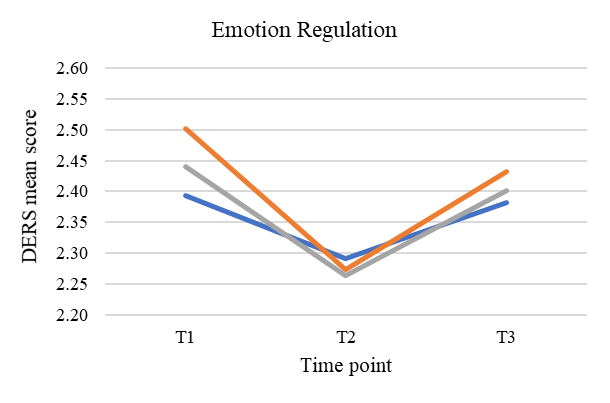


**Figure S1** Variable mean scores across time and group. T1 = baseline, T2 = 2 months following baseline, T3 = 7 months following baseline.


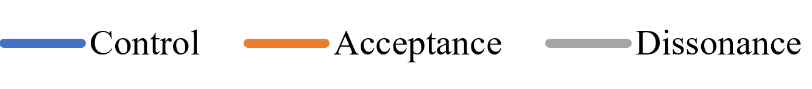


**Figure S2.** Mediation models for sensitivity analysis (a) using listwise deletion to manage all missing data: emotion regulation as a mediator of the relationship between mindfulness and weight and shape concerns (top panel), controlling for previous levels of the mediator and outcome (bottom panel). T1 = baseline, T2 = 2 months following baseline, T3 = 7 months following baseline. Coefficients are unstandardised. Values in square brackets are 95% CIs. c = X–Y total effect, c' = X–Y direct effect. ^***^p < .001. ^**^p < .01. ^*^p < .05.


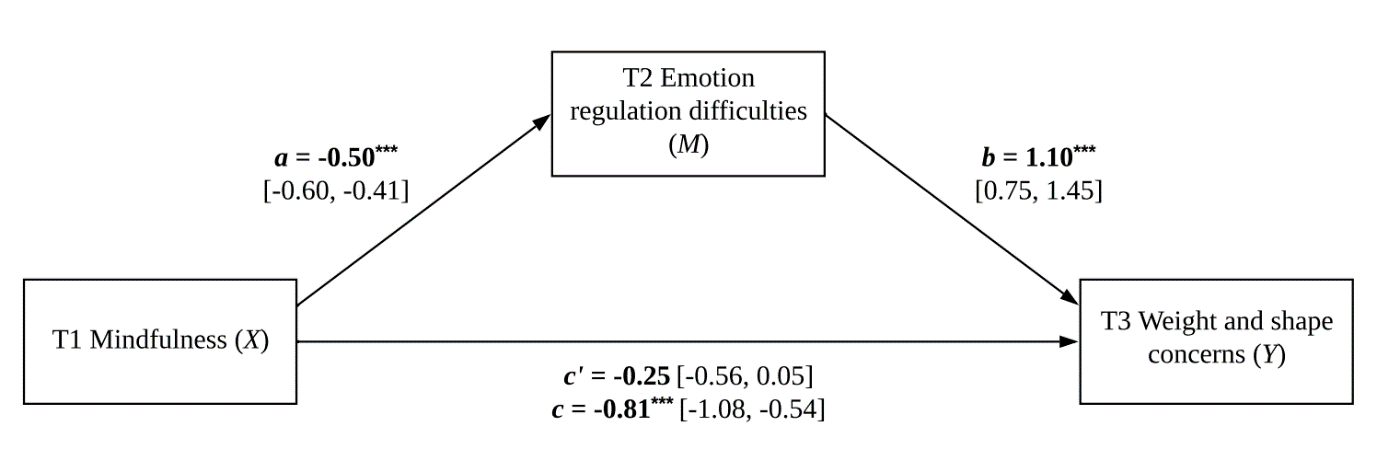

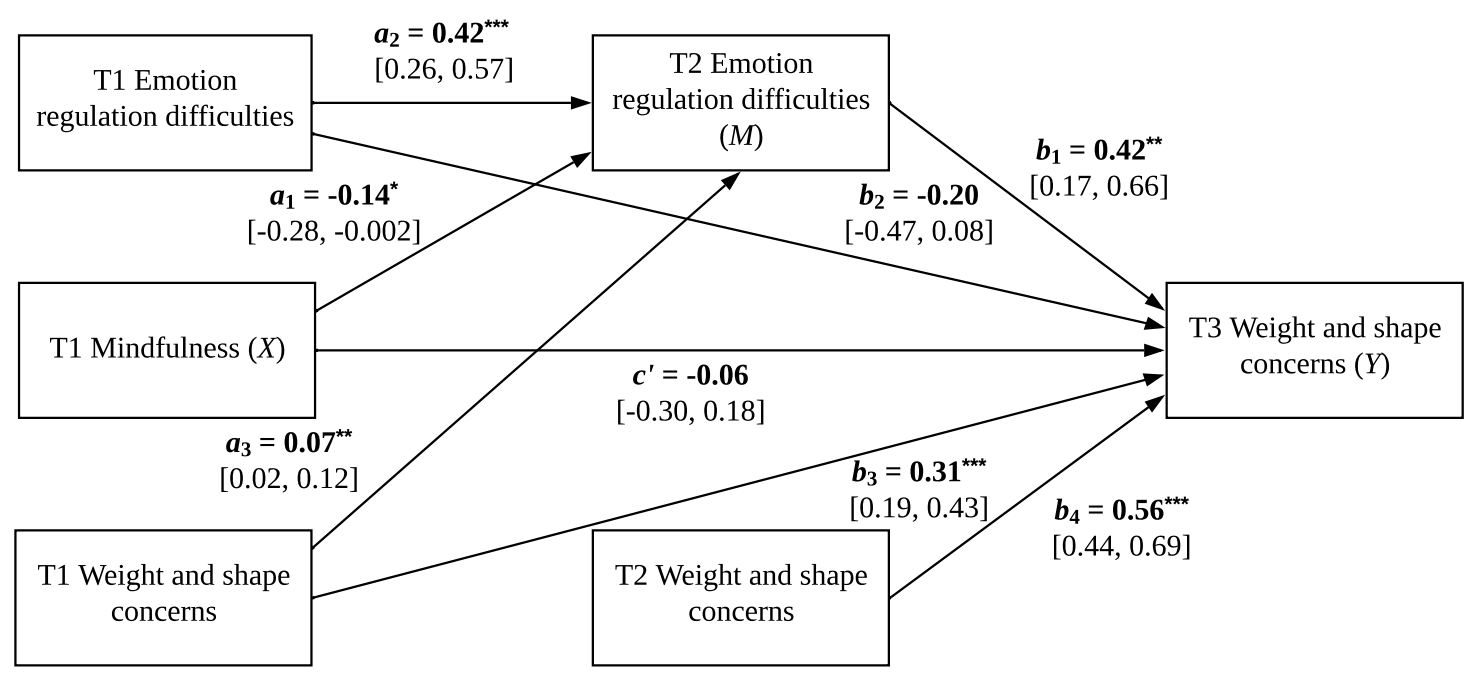


**Figure S3.** Mediation models for sensitivity analysis (a) using listwise deletion to manage all missing data: emotion regulation as a mediator of the relationship between mindfulness and negative affect (top panel), controlling for previous levels of the mediator and outcome (bottom panel). T1 = baseline, T2 = 2 months following baseline, T3 = 7 months following baseline. Coefficients are unstandardised. Values in square brackets are 95% CIs. c = X–Y total effect, c' = X–Y direct effect. ^***^p < .001. ^**^p < .01. ^*^p < .05.


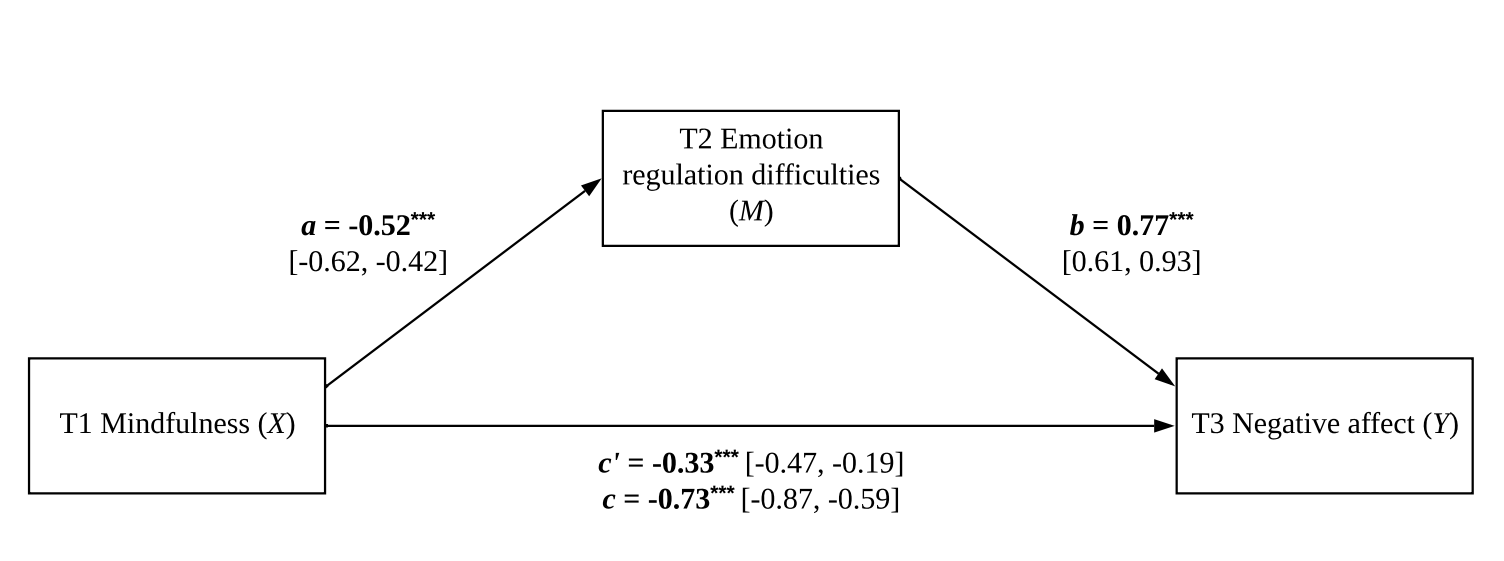

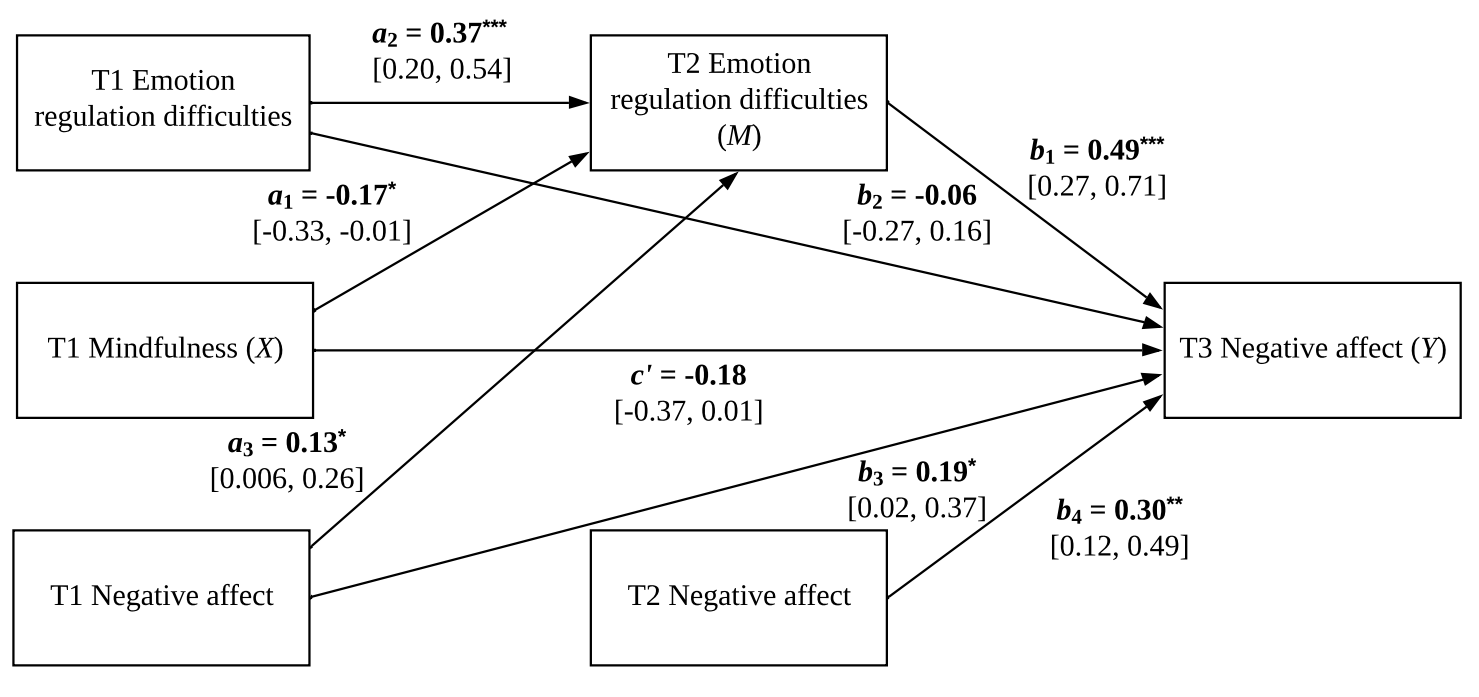


# Tables

**Table S1**

Differences between groups across variables and timepoints.

|  |  | *N* | *M* | *SD* | 95% CI | | *F*(2, 371) | *p* |
| --- | --- | --- | --- | --- | --- | --- | --- | --- |
|  |  |  |  |  | LL | UL |  |  |
| T1 Mindfulness | Control | 112 | 3.71 | 0.73 | 3.58 | 3.85 | 1.02 | .361 |
|  | Acceptance | 141 | 3.57 | 0.88 | 3.42 | 3.71 |  |  |
|  | Dissonance | 121 | 3.63 | 0.76 | 3.49 | 3.77 |  |  |
|  | Total | 374 | 3.63 | 0.80 | 3.55 | 3.71 |  |  |
| T2 Mindfulness | Control | 112 | 3.89 | 0.83 | 3.73 | 4.04 | 0.01 | .987 |
|  | Acceptance | 141 | 3.90 | 0.85 | 3.75 | 4.04 |  |  |
|  | Dissonance | 121 | 3.88 | 0.78 | 3.74 | 4.02 |  |  |
|  | Total | 374 | 3.89 | 0.82 | 3.80 | 3.97 |  |  |
| T3 Mindfulness | Control | 112 | 3.78 | 0.89 | 3.61 | 3.94 | 0.17 | .848 |
|  | Acceptance | 141 | 3.83 | 0.94 | 3.67 | 3.99 |  |  |
|  | Dissonance | 121 | 3.78 | 0.81 | 3.63 | 3.92 |  |  |
|  | Total | 374 | 3.80 | 0.88 | 3.71 | 3.89 |  |  |
| T1 Emotion regulation difficulties | Control | 112 | 2.39 | 0.72 | 2.26 | 2.53 | 0.74 | .479 |
|  | Acceptance | 141 | 2.50 | 0.76 | 2.38 | 2.63 |  |  |
|  | Dissonance | 121 | 2.44 | 0.69 | 2.32 | 2.56 |  |  |
|  | Total | 374 | 2.45 | 0.72 | 2.38 | 2.52 |  |  |
| T2 Emotion regulation difficulties | Control | 112 | 2.29 | 0.71 | 2.16 | 2.42 | 0.04 | .957 |
|  | Acceptance | 141 | 2.27 | 0.73 | 2.15 | 2.39 |  |  |
|  | Dissonance | 121 | 2.26 | 0.67 | 2.14 | 2.38 |  |  |
|  | Total | 374 | 2.28 | 0.70 | 2.20 | 2.35 |  |  |
| T3 Emotion regulation difficulties | Control | 112 | 2.38 | 0.80 | 2.23 | 2.53 | 0.14 | .873 |
|  | Acceptance | 141 | 2.43 | 0.83 | 2.29 | 2.57 |  |  |
|  | Dissonance | 121 | 2.40 | 0.70 | 2.28 | 2.53 |  |  |
|  | Total | 374 | 2.41 | 0.78 | 2.33 | 2.49 |  |  |
| T1 Weight and shape concerns | Control | 112 | 2.68 | 1.74 | 2.36 | 3.01 | 1.37 | .255 |
|  | Acceptance | 141 | 2.85 | 1.66 | 2.57 | 3.12 |  |  |
|  | Dissonance | 121 | 2.50 | 1.63 | 2.21 | 2.80 |  |  |
|  | Total | 374 | 2.69 | 1.68 | 2.52 | 2.86 |  |  |
| T2 Weight and shape concerns | Control | 112 | 2.38 | 1.75 | 2.05 | 2.71 | 1.53 | .219 |
|  | Acceptance | 141 | 2.31 | 1.71 | 2.02 | 2.59 |  |  |
|  | Dissonance | 121 | 2.01 | 1.65 | 1.72 | 2.31 |  |  |
|  | Total | 374 | 2.23 | 1.71 | 2.06 | 2.41 |  |  |
| T3 Weight and shape concerns | Control | 112 | 2.50 | 1.86 | 2.15 | 2.84 | 1.17 | .312 |
|  | Acceptance | 141 | 2.50 | 1.79 | 2.20 | 2.80 |  |  |
|  | Dissonance | 121 | 2.20 | 1.57 | 1.92 | 2.48 |  |  |
|  | Total | 374 | 2.40 | 1.75 | 2.22 | 2.58 |  |  |
| T1 Negative affect | Control | 112 | 2.19 | 0.83 | 2.03 | 2.34 | 0.29 | .752 |
|  | Acceptance | 141 | 2.20 | 0.89 | 2.05 | 2.35 |  |  |
|  | Dissonance | 121 | 2.12 | 0.83 | 1.98 | 2.27 |  |  |
|  | Total | 374 | 2.17 | 0.85 | 2.09 | 2.26 |  |  |
| T2 Negative affect | Control | 112 | 2.21 | 0.92 | 2.03 | 2.38 | 0.76 | .470 |
|  | Acceptance | 141 | 2.10 | 0.94 | 1.95 | 2.26 |  |  |
|  | Dissonance | 121 | 2.06 | 0.91 | 1.90 | 2.23 |  |  |
|  | Total | 374 | 2.12 | 0.92 | 2.03 | 2.22 |  |  |
| T3 Negative affect | Control | 112 | 2.28 | 0.88 | 2.12 | 2.44 | 0.86 | .423 |
|  | Acceptance | 141 | 2.29 | 0.94 | 2.13 | 2.45 |  |  |
|  | Dissonance | 121 | 2.15 | 0.89 | 1.99 | 2.31 |  |  |
|  | Total | 374 | 2.24 | 0.91 | 2.15 | 2.33 |  |  |
| *Note*. We used variables after data cleaning. T1 = baseline, T2 = 2 months following baseline, T3 = 7 months following baseline. 95% CI = 95% confidence interval for the mean. LL = Lower limit. UL = Upper limit. | | | | | | | | |

**Table S2**

Absolute values of skewness and kurtosis.

|  | Skewness | | Kurtosis | |
| --- | --- | --- | --- | --- |
|  | Statistic | *SE* | Statistic | *SE* |
| T1 Mindfulness | -0.58 | 0.13 | -0.39 | 0.25 |
| T1 Emotion regulation difficulties | 0.55 | 0.13 | -0.44 | 0.25 |
| T2 Emotion regulation difficulties | 0.57 | 0.13 | -0.33 | 0.25 |
| T1 Weight and shape concerns | 0.22 | 0.13 | -1.16 | 0.25 |
| T2 Weight and shape concerns | 0.44 | 0.13 | -1.01 | 0.25 |
| T3 Weight and shape concerns | 0.41 | 0.13 | -0.96 | 0.25 |
| T1 Negative affect | 0.69 | 0.13 | -0.60 | 0.25 |
| T2 Negative affect | 0.75 | 0.13 | -0.46 | 0.25 |
| T3 Negative affect | 0.66 | 0.13 | -0.25 | 0.25 |
| T1 = baseline, T2 = 2 months following baseline, T3 = 7 months following baseline. | | | | |

**Table S3**

Univariate outliers.

|  | Range for outliers | | Outliers | | |
| --- | --- | --- | --- | --- | --- |
|  | LL | UL | No. | Values | Winsorized |
| T1 Mindfulness | 1.78 | 5.62 | 12 | 1.00, 1.00, 1.00, 1.20, 1.20, 1.20, 1.30, 1.40, 1.50, 1.60, 1.70, 1.72 | 1.80 |
| T1 Emotion regulation difficulties | 0.58 | 4.01 | 13 | 5.00, 4.56, 4.56, 4.50, 4.39, 4.33, 4.22, 4.22, 4.11, 4.11, 4.06, 4.06, 4.06 | 4.00 |
| T2 Emotion regulation difficulties | 0.42 | 3.99 | 10 | 5.00, 4.72, 4.72, 4.50, 4.33, 4.17, 4.17, 4.11, 4.00, 4.00 | 3.93 |
| T1 Weight and shape concerns | -3.83 | 6.67 | 0 | – | – |
| T2 Weight and shape concerns | -3.03 | 6.86 | 0 | – | – |
| T3 Weight and shape concerns | -2.94 | 7.10 | 0 | – | – |
| T1 Negative affect | -0.21 | 4.08 | 14 | 4.94, 4.88, 4.88, 4.82, 4.59, 4.53, 4.50, 4.47, 4.41, 4.41, 4.35, 4.29, 4.18, 4.13 | 4.06 |
| T2 Negative affect | -0.52 | 4.28 | 14 | 5.00, 5.00, 5.00, 5.00, 5.00, 4.94, 4.88, 4.82, 4.76, 4.47, 4.47, 4.41, 4.32, 4.29 | 4.24 |
| T3 Negative affect | -0.43 | 4.61 | 6 | 5.00, 5.00, 4.88, 4.71, 4.71, 4.65 | 4.59 |
| T1 = baseline, T2 = 2 months following baseline, T3 = 7 months following baseline. LL = Lower limit. UL = Upper limit. No. = Number of outliers. | | | | | |

**Table S4**

Statistics for testing independent errors and multicollinearity.

| Outcome | Model | D–W value | VIF | Tolerance |
| --- | --- | --- | --- | --- |
| Weight and shape concerns | Without covariates | 2.04 | 1.49 | 0.67 |
|  | Lags as covariates | 1.83 | 2.29–3.01 | 0.33–0.44 |
| Negative affect | Without covariates | 1.77 | 1.49 | 0.67 |
|  | Lags as covariates | 1.80 | 2.50–3.33 | 0.30–0.40 |
| D–W value = Durbin–Watson statistic. VIF = Variance inflation factor. | | | | |

**Table S5**

Bivariate correlations between BMI, age, and variables included in the mediation models.

|  | Mindfulness | Emotion regulation difficulties | | Weight and shape concerns | | | Negative affect | | |
| --- | --- | --- | --- | --- | --- | --- | --- | --- | --- |
|  | T1 | T1 | T2 | T1 | T2 | T3 | T1 | T2 | T3 |
| BMI | -.14^*^ | .12^*^ | .07 | .36^***^ | .30^***^ | .28^***^ | .11 | .07 | .18^**^ |
| Age | -.01 | .03 | -.07 | .02 | .002 | .01 | .06 | -.04 | -.002 |

T1 = baseline, T2 = 2 months following baseline, T3 = 7 months following baseline. ^***^*p* < .001. ^**^*p* < .01. ^*^*p* < .05.

**Table S6**

Path estimates in the model of emotion regulation difficulties as mediator of the relationship between mindfulness and weight and shape concerns (top panel), using earlier measurements (aka “lags”) as covariates (bottom panel).

|  |  | | Consequent | | | | | | | | | | | | |
| --- | --- | --- | --- | --- | --- | --- | --- | --- | --- | --- | --- | --- | --- | --- | --- |
|  |  | | T2 Emotion regulation difficulties (*M*) | | | | | |  | | T3 Weight and shape concerns (*Y*) | | | | |
| Antecedent |  | | Coeff. | | *SE* | | *p* | |  | | Coeff. | | *SE* | | *p* |
| T1 Mindfulness (*X*) | *a* | | -0.50 [-0.58, -0.43] | | 0.04 | | <.001 | | *c* | | -0.76 [-0.97, -0.56] | | 0.11 | | <.001 |
|  |  |  |  |  |  |  |  |  | *c'* | | -0.25 [-0.49, -0.01] | | 0.12 | | .042 |
| T2 Emotion regulation difficulties (*M*) |  | | – | | – | | – | | *b* | | 1.03 [0.76, 1.30] | | 0.14 | | <.001 |
|  |  | | *R*^2^ = .328 | | | | | |  | | *R*^2^ = .237 | | | | |
|  |  | | *F*(1, 372) = 181.84, *p <* .001 | | | | | |  | | *F*(2, 371) = 57.44, *p* < .001 | | | | |
| T1 Mindfulness (*X*) | | *a*_1_ | | -0.08 [-0.18, 0.01] | | 0.05 | | .083 | | *c'* | | -0.07 [-0.23, 0.10] | | 0.08 | .418 |
| T1 Emotion regulation difficulties | | *a*_2_ | | 0.55 [0.44, 0.66] | | 0.05 | | <.001 | | *b*_2_ | | -0.19 [-0.39, 0.02] | | 0.11 | .077 |
| T1 Weight and shape concerns | | *a*_3_ | | 0.06 [0.03, 0.09] | | 0.02 | | <.001 | | *b*_3_ | | 0.29 [0.20, 0.38] | | 0.04 | <.001 |
| T2 Emotion regulation difficulties (*M*) | |  | | – | | – | | – | | *b*_1_ | | 0.21 [0.02, 0.39] | | 0.09 | .028 |
| T2 Weight and shape concerns | |  | |  | |  | |  | | *b*_4_ | | 0.63 [0.55, 0.72] | | 0.04 | <.001 |
|  | |  | | *R*^2^ = .514 | | | | | |  | | *R*^2^ = .771 | | | |
|  | |  | | *F*(3, 368) = 129.78, *p <* .001 | | | | | |  | | *F*(5, 366) = 246.93, *p* < .001 | | | |

T1 = baseline, T2 = 2 months following baseline, T3 = 7 months following baseline. a = X–M, b = M–Y, c = X–Y total effect, c' = X–Y direct effect. Coefficients are unstandardised. Values in square brackets are 95% CIs.

**Table S7**

Path estimates in the model of emotion regulation difficulties as mediator of the relationship between mindfulness and negative affect (top panel), using earlier measurements (aka “lags”) as covariates (bottom panel).

|  |  | | Consequent | | | | | | | | | | | | |  |
| --- | --- | --- | --- | --- | --- | --- | --- | --- | --- | --- | --- | --- | --- | --- | --- | --- |
|  |  | | T2 Emotion regulation difficulties (*M*) | | | | | |  | | T3 Negative affect (*Y*) | | | | |  |
| Antecedent |  | | Coeff. | | *SE* | | *p* | |  | | Coeff. | | *SE* | | *p* |  |
| T1 Mindfulness (*X*) | *a* | | -0.50 [-0.57, -0.42] | | 0.04 | | <.001 | | *c* | | -0.64 [-0.74, -0.54] | | 0.05 | | <.001 |  |
|  |  |  |  |  |  |  |  |  | *c'* | | -0.28 [-0.38, -0.18] | | 0.05 | | <.001 |  |
| T2 Emotion regulation difficulties (*M*) |  | | – | | – | | – | | *b* | | 0.72 [0.61, 0.83] | | 0.06 | | <.001 |  |
|  |  | | *R*^2^ = .321 | | | | | |  | | *R*^2^ = .524 | | | | |  |
|  |  | | *F*(1, 371) = 175.64, *p <*.001 | | | | | |  | | *F*(2, 370) = 203.90, *p* < .001 | | | | |  |
| T1 Mindfulness (*X*) | | *a*_1_ | | -0.10 [-0.20, -0.003] | | 0.05 | | .043 | | *c'* | | -0.12 [-0.24, -0.007] | | 0.06 | .038 | |
| T1 Emotion regulation difficulties | | *a*_2_ | | 0.51 [0.40, 0.63] | | 0.06 | | <.001 | | *b*_2_ | | 0.05 [-0.09, 0.20] | | 0.07 | .466 | |
| T1 Negative affect | | *a*_3_ | | 0.12 [0.03, 0.21] | | 0.04 | | .007 | | *b*_3_ | | 0.12 [0.006, 0.24] | | 0.06 | .039 | |
| T2 Emotion regulation difficulties (*M*) | |  | | – | | – | | – | | *b*_1_ | | 0.32 [0.18, 0.46] | | 0.07 | <.001 | |
| T2 Negative affect | |  | | – | | – | | – | | *b*_4_ | | 0.40 [0.29, 0.51] | | 0.06 | <.001 | |
|  | |  | | *R*^2^ = .526 | | | | | |  | | *R*^2^ = .627 | | | | |
|  | |  | | *F*(3, 365) = 134.97, *p <* .001 | | | | | |  | | *F*(5, 363) = 122.15, *p* < .001 | | | | |

T1 = baseline, T2 = 2 months following baseline, T3 = 7 months following baseline. a = X–M, b = M–Y, c = X–Y total effect, c' = X–Y direct effect. Coefficients are unstandardised. Values in square brackets are 95% CIs.

**Table S8**

Path estimates in the model of emotion regulation difficulties as mediator of the relationship between mindfulness and weight and shape concerns (top panel), using earlier measurements (aka “lags”) as covariates (bottom panel).

|  |  | | Consequent | | | | | | | | | | | | |
| --- | --- | --- | --- | --- | --- | --- | --- | --- | --- | --- | --- | --- | --- | --- | --- |
|  |  | | T2 Emotion regulation difficulties (*M*) | | | | | |  | | T3 Weight and shape concerns (*Y*) | | | | |
| Antecedent |  | | Coeff. | | *SE* | | *p* | |  | | Coeff. | | *SE* | | *p* |
| T1 Mindfulness (*X*) | *a* | | -0.50 [-0.60, -0.41] | | 0.05 | | <.001 | | *c* | | -0.81 [-1.08, -0.54] | | 0.14 | | <.001 |
|  |  |  |  |  |  |  |  |  | *c'* | | -0.25 [-0.56, 0.05] | | 0.16 | | .105 |
| T2 Emotion regulation difficulties (*M*) |  | | – | | – | | – | | *b* | | 1.10 [0.75, 1.45] | | 0.18 | | <.001 |
|  |  | | *R*^2^ = .330 | | | | | |  | | *R*^2^ = .257 | | | | |
|  |  | | *F*(1, 228) = 112.16, *p <* .001 | | | | | |  | | *F*(2, 227) = 39.31, *p* < .001 | | | | |
| T1 Mindfulness (*X*) | | *a*_1_ | | -0.14 [-0.28, -0.002] | | 0.07 | | .047 | | *c'* | | -0.06 [-0.30, 0.18] | | 0.12 | .618 |
| T1 Emotion regulation difficulties | | *a*_2_ | | 0.42 [0.26, 0.57] | | 0.08 | | <.001 | | *b*_2_ | | -0.20 [-0.47, 0.08] | | 0.14 | .164 |
| T1 Weight and shape concerns | | *a*_3_ | | 0.07 [0.02, 0.12] | | 0.02 | | .003 | | *b*_3_ | | 0.31 [0.19, 0.43] | | 0.06 | <.001 |
| T2 Emotion regulation difficulties (*M*) | |  | | – | | – | | – | | *b*_1_ | | 0.42 [0.17, 0.66] | | 0.12 | .001 |
| T2 Weight and shape concerns | |  | |  | |  | |  | | *b*_4_ | | 0.56 [0.44, 0.69] | | 0.06 | <.001 |
|  | |  | | *R*^2^ = .458 | | | | | |  | | *R*^2^ = .762 | | | |
|  | |  | | *F*(3, 209) = 58.96, *p <* .001 | | | | | |  | | *F*(5, 207) = 132.48, *p* < .001 | | | |

Sensitivity analysis (a) using listwise deletion. T1 = baseline, T2 = 2 months following baseline, T3 = 7 months following baseline. a = X–M, b = M–Y, c = X–Y total effect, c' = X–Y direct effect. Coefficients are unstandardised. Values in square brackets are 95% CIs.

**Table S9**

Path estimates in the model of emotion regulation difficulties as mediator of the relationship between mindfulness and negative affect (top panel), using earlier measurements (aka “lags”) as covariates (bottom panel).

|  |  | | Consequent | | | | | | | | | | | | |
| --- | --- | --- | --- | --- | --- | --- | --- | --- | --- | --- | --- | --- | --- | --- | --- |
|  |  | | T2 Emotion regulation difficulties (*M*) | | | | | |  | | T3 Negative affect (*Y*) | | | | |
| Antecedent |  | | Coeff. | | *SE* | | *p* | |  | | Coeff. | | *SE* | | *p* |
| T1 Mindfulness (*X*) | *a* | | -0.52 [-0.62, -0.42] | | 0.05 | | <.001 | | *c* | | -0.73 [-0.87, -0.59] | | 0.07 | | <.001 |
|  |  |  |  |  |  |  |  |  | *c'* | | -0.33 [-0.47, -0.19] | | 0.07 | | <.001 |
| T2 Emotion regulation difficulties (*M*) |  | | – | | – | | – | | *b* | | 0.77 [0.61, 0.93] | | 0.08 | | <.001 |
|  |  | | *R*^2^ = .331 | | | | | |  | | *R*^2^ = .523 | | | | |
|  |  | | *F*(1, 223) = 110.24, *p <*.001 | | | | | |  | | *F*(2, 222) = 121.49, *p* < .001 | | | | |
| T1 Mindfulness (*X*) | | *a*_1_ | | -0.17 [-0.33, -0.01] | | 0.08 | | .038 | | *c'* | | -0.18 [-0.37, 0.01] | | 0.10 | .069 |
| T1 Emotion regulation difficulties | | *a*_2_ | | 0.37 [0.20, 0.54] | | 0.09 | | <.001 | | *b*_2_ | | -0.06 [-0.27, 0.16] | | 0.11 | .594 |
| T1 Negative affect | | *a*_3_ | | 0.13 [0.006, 0.26] | | 0.06 | | .040 | | *b*_3_ | | 0.19 [0.02, 0.37] | | 0.09 | .033 |
| T2 Emotion regulation difficulties (*M*) | |  | | – | | – | | – | | *b*_1_ | | 0.49 [0.27, 0.71] | | 0.11 | <.001 |
| T2 Negative affect | |  | | – | | – | | – | | *b*_4_ | | 0.30 [0.12, 0.49] | | 0.09 | .001 |
|  | |  | | *R*^2^ = .445 | | | | | |  | | *R*^2^ = .603 | | | |
|  | |  | | *F*(3, 200) = 53.40, *p <* .001 | | | | | |  | | *F*(5, 198) = 60.05, *p* < .001 | | | |

Sensitivity analysis (a) using listwise deletion. T1 = baseline, T2 = 2 months following baseline, T3 = 7 months following baseline. a = X–M, b = M–Y, c = X–Y total effect, c' = X–Y direct effect. Coefficients are unstandardised. Values in square brackets are 95% CIs.

**Table S10**

Test statistics for differences between participants who were included compared to excluded in the second sensitivity analysis (i.e., Scenario 2).

|  | *M* (*SD*) | | *t* | *df* | *p* |
| --- | --- | --- | --- | --- | --- |
|  | Included | Excluded |  |  |  |
| Age | 15.65 (0.74) | 15.82 (0.83) | 2.04 | 372 | .042 |
| BMI | 20.76 (2.88) | 20.74 (3.05) | -0.05 | 304 | .958 |
| Mindfulness | 3.65 (0.84) | 3.55 (0.90) | -0.94 | 338 | .349 |
| Emotion regulation difficulties | 2.42 (0.76) | 2.51 (0.79) | 0.90 | 324 | .367 |
| Weight and shape concerns | 2.59 (1.74) | 3.01 (1.57) | 1.95 | 339 | .052 |
| Negative affect | 2.14 (0.90) | 2.32 (0.95) | 1.63 | 329 | .104 |
| *Note*. Variables at baseline.  Included = participants who were present at all three waves of measurement.  Excluded = participants who were absent for one or more waves of measurement.  Bonferroni corrected *α* = .05/6 = .0083. | | | | | |

# References

Berg, K. C., Peterson, C. B., Frazier, P., & Crow, S. J. (2012). Psychometric evaluation of the eating disorder examination and eating disorder examination-questionnaire: A systematic review of the literature. *International Journal of Eating Disorders*, *45*(3), 428–438. https://doi.org/10.1002/eat.20931

Cox, B. E., McIntosh, K., Reason, R. D., & Terenzini, P. T. (2014). Working with missing data in higher education research: A primer and real-world example. *Review of Higher Education, 37*(3), 377–402. https://doi.org/10.1353/rhe.2014.0026

Fairburn, C. G., & Beglin, S. J. (1994). Assessment of eating disorders: Interview or self‐report questionnaire? *International Journal of Eating Disorders*, *16*(4), 363–370.

Field, A. (2013). *Discovering statistics using IBM SPSS statistics: And sex and drugs and rock’n’roll* (4th ed.). SAGE.

Gall, K., Van Zutven, K., Lindstrom, J., Bentley, C., Gratwick-Sarll, K., Harrison, C., Lewis, V., & Mond, J. (2016). Obesity and emotional well-being in adolescents: Roles of body dissatisfaction, loss of control eating, and self-rated health. *Obesity*, *24*(4), 837–842. https://doi.org/10.1002/oby.21428

Greco, L. A., Baer, R. A., & Smith, G. T. (2011). Assessing mindfulness in children and adolescents: Development and validation of the child and adolescent mindfulness measure (CAMM). *Psychological Assessment*, *23*(3), 606–614. https://doi.org/10.1037/a0022819

Hayes, A. F. (2022). *Introduction to mediation, moderation, and conditional process analysis: A regression-based approach* (3rd ed.). Guilford Press.

International Journal of Eating Disorders. (2019). IJED statistical reporting guidelines. https://onlinelibrary.wiley.com/pb-assets/assets/1098108X/IJED_Statistical_Reporting_Guidelines_revised_April_2019-1557345377963.pdf

Kaufman, E. A., Xia, M., Fosco, G., Yaptangco, M., Skidmore, C. R., & Crowell, S. E. (2016). The Difficulties in Emotion Regulation Scale Short Form (DERS-SF): Validation and replication in adolescent and adult samples. *Journal of Psychopathology and Behavioral Assessment*, *38*(3), 443–455. https://doi.org/10.1007/s10862-015-9529-3

Kim, H.-Y. (2013). Statistical notes for clinical researchers: Assessing normal distribution (2) using skewness and kurtosis. *Restorative Dentistry & Endodontics*, *38*(1), 52. https://doi.org/10.5395/rde.2013.38.1.52

Leys, C., Ley, C., Klein, O., Bernard, P., & Licata, L. (2013). Detecting outliers: Do not use standard deviation around the mean, use absolute deviation around the median. *Journal of Experimental Social Psychology*, *49*(4), 764–766. https://doi.org/10.1016/j.jesp.2013.03.013

Mackinnon, D. P., Lockwood, C. M., & Williams, J. (2004). Confdence limits for the indirect effect: Distribution of the product and resampling methods. *Multivariate Behavioral Research, 39*(1), 99–128. https://doi.org/10.1207/s15327906mbr3901_4

Menard, S. (1995). *Applied logistic regression analysis (Sage university paper series on quantitative application in the social sciences, series no. 106)* (2nd ed.). Sage.

Mond, J. M., Hall, A., Bentley, C., Harrison, C., Gratwick-Sarll, K., & Lewis, V. (2014). Eating-disordered behavior in adolescent boys: Eating disorder examination questionnaire norms. *International Journal of Eating Disorders*, *47*(4), 335–341. https://doi.org/10.1002/eat.22237

Mond, J. M., Rodgers, B., Hay, P. J., Darby, A., Owen, C., Baune, B. T., & Kennedy, R. L. (2007). Obesity and impairment in psychosocial functioning in women: The mediating role of eating disorder features. *Obesity*, *15*(11), 2769–2779. https://doi.org/10.1038/oby.2007.329

Myers, R. (1990). *Classical and modern regression with applications* (2nd ed.). Duxbury.

Rand-Giovannetti, D., Cicero, D. C., Mond, J. M., & Latner, J. D. (2020). Psychometric properties of the Eating Disorder Examination–Questionnaire (EDE-Q): A confirmatory factor analysis and assessment of measurement invariance by sex. *Assessment*, *27*(1), 164–177. https://doi.org/10.1177/1073191117738046

Reifman, A., & Keyton, K. (2012). Winsorize. In N. J. Salkind (Ed.), *Encyclopedia of Research Design*. Sage. https://doi.org/10.4135/9781412961288

Scheffer, J. (2002). Dealing with missing data. *Res. Lett. Inf. Math. Sci*., *3*, 153–160.

Stice, E., & Agras, W. S. (1998). Predicting onset and cessation of bulimic behaviors during adolescence: A longitudinal grouping analysis. *Behavior Therapy*, *29*(2), 257–276. https://doi.org/10.1016/S0005-7894(98)80006-3

Tabachnick, B. G., & Fidell, L. S. (2019). *Using multivariate statistics* (7th ed.). Pearson.

Trompeter, N., Bussey, K., Forbes, M. K., Hay, P., Goldstein, M., Thornton, C., Basten, C., Heruc, G., Roberts, M., Byrne, S., Griffiths, S., Lonergan, A., & Mitchison, D. (2022). Emotion dysregulation and eating disorder symptoms: Examining distinct associations and interactions in adolescents. *Research on Child and Adolescent Psychopathology*, *50*, 683–694. https://doi.org/10.1007/s10802-022-00898-1

Van Zutven, K., Mond, J. M., Latner, J., & Rodgers, B. (2015). Obesity and psychosocial impairment: Mediating roles of health status, weight/shape concerns and binge eating in a community sample of women and men. *International Journal of Obesity*, *39*(2), 346–352. https://doi.org/10.1038/ijo.2014.100

Watson, D., & Clark, L. A. (1994). *The PANAS-X: Manual for the Positive and Negative Effect Schedule - Expanded Form*. University of Iowa.

Wilksch, S. M., & Wade, T. D. (2009). Reduction of shape and weight concern in young adolescents: A 30-month controlled evaluation of a media literacy program. *Journal of the American Academy of Child and Adolescent Psychiatry*, *48*(6), 652–661. https://doi.org/10.1097/CHI.0b013e3181a1f559

# Appendix A

Details on the detection and treatment of univariate outliers in the first sensitivity analysis.

(1) Model of weight and shape concerns without covariates:

Variable: T1 MIFU

MAD: 0.831903333333333

Median: 3.738888888888889

Lowerbound: 1.6591305555555564

Upperbound: 5.818647222222221

Winsorization done

new old higher/lower_bound

2 1.7 1.5 lower

15 1.7 1.4 lower

44 1.7 1.6 lower

51 1.7 1.0 lower

62 1.7 1.2 lower

83 1.7 1.2 lower

93 1.7 1.0 lower

107 1.7 1.2 lower

Number winsorized to lower bound: 8

Number winsorized to upper bound: 0

Variable: T2 ER

MAD: 0.6589333333333333

Median: 2.111111111111111

Lowerbound: 0.46377777777777807

Upperbound: 3.7584444444444443

Winsorization done

new old higher/lower_bound

13 3.722222 3.777778 upper

33 3.722222 3.777778 upper

42 3.722222 3.777778 upper

48 3.722222 3.833333 upper

60 3.722222 4.333333 upper

87 3.722222 4.722222 upper

92 3.722222 5.000000 upper

93 3.722222 4.722222 upper

95 3.722222 3.777778 upper

103 3.722222 4.111111 upper

107 3.722222 4.166667 upper

Number winsorized to lower bound: 0

Number winsorized to upper bound: 11

Variable: T3 WSC

MAD: 1.8532499999999998

Median: 1.75

Lowerbound: -2.8831249999999997

Upperbound: 6.383125

no winsorization needed!

(2) Model of negative affect without covariates:

Variable: T1 MIFU

MAD: 0.7742466666666665

Median: 3.7777777777777777

Lowerbound: 1.8421611111111114

Upperbound: 5.713394444444444

Winsorization done

new old higher/lower_bound

2 2.0 1.5 lower

15 2.0 1.4 lower

44 2.0 1.6 lower

51 2.0 1.0 lower

62 2.0 1.2 lower

83 2.0 1.2 lower

92 2.0 1.7 lower

93 2.0 1.0 lower

107 2.0 1.2 lower

Number winsorized to lower bound: 9

Number winsorized to upper bound: 0

Variable: T2 ER

MAD: 0.6589333333333333

Median: 2.111111111111111

Lowerbound: 0.46377777777777807

Upperbound: 3.7584444444444443

Winsorization done

new old higher/lower_bound

13 3.722222 3.777778 upper

33 3.722222 3.777778 upper

42 3.722222 3.777778 upper

48 3.722222 3.833333 upper

60 3.722222 4.333333 upper

87 3.722222 4.722222 upper

92 3.722222 5.000000 upper

93 3.722222 4.722222 upper

103 3.722222 4.111111 upper

107 3.722222 4.166667 upper

Number winsorized to lower bound: 0

Number winsorized to upper bound: 10

Variable: T3 NA

MAD: 1.046541176470588

Median: 1.9411764705882353

Lowerbound: -0.6751764705882348

Upperbound: 4.557529411764706

Winsorization done

new old higher/lower_bound

38 4.529412 4.705882 upper

42 4.529412 5.000000 upper

62 4.529412 4.882353 upper

87 4.529412 4.705882 upper

92 4.529412 5.000000 upper

93 4.529412 4.588235 upper

103 4.529412 4.647059 upper

Number winsorized to lower bound: 0

Number winsorized to upper bound: 7

(3) Model of weight and shape concerns using earlier measurements (aka “lags”) as covariates:

Variable: T1 MIFU

MAD: 0.8730866666666668

Median: 3.7

Lowerbound: 1.5172833333333333

Upperbound: 5.882716666666667

Winsorization done

new old higher/lower_bound

2 1.6 1.5 lower

51 1.6 1.0 lower

62 1.6 1.2 lower

83 1.6 1.2 lower

93 1.6 1.0 lower

107 1.6 1.2 lower

Number winsorized to lower bound: 6

Number winsorized to upper bound: 0

Variable: T1 ER

MAD: 0.7413

Median: 2.2777777777777777

Lowerbound: 0.42452777777777784

Upperbound: 4.131027777777778

Winsorization done

new old higher/lower_bound

48 4.111111 4.388889 upper

51 4.111111 4.500000 upper

59 4.111111 4.333333 upper

60 4.111111 4.555556 upper

92 4.111111 5.000000 upper

103 4.111111 4.222222 upper

Number winsorized to lower bound: 0

Number winsorized to upper bound: 6

Variable: T2 ER

MAD: 0.6589333333333333

Median: 2.111111111111111

Lowerbound: 0.46377777777777807

Upperbound: 3.7584444444444443

Winsorization done

new old higher/lower_bound

13 3.722222 3.777778 upper

33 3.722222 3.777778 upper

48 3.722222 3.833333 upper

60 3.722222 4.333333 upper

87 3.722222 4.722222 upper

92 3.722222 5.000000 upper

93 3.722222 4.722222 upper

95 3.722222 3.777778 upper

103 3.722222 4.111111 upper

107 3.722222 4.166667 upper

Number winsorized to lower bound: 0

Number winsorized to upper bound: 10

Variable: T1 WSC

MAD: 1.9768000000000001

Median: 2.0833333333333335

Lowerbound: -2.8586666666666667

Upperbound: 7.025333333333334

no winsorization needed!

Variable: T2 WSC

MAD: 1.7297

Median: 1.5833333333333333

Lowerbound: -2.740916666666667

Upperbound: 5.907583333333333

Winsorization done

new old higher/lower_bound

51 5.833333 6.0 upper

92 5.833333 6.0 upper

123 5.833333 6.0 upper

Number winsorized to lower bound: 0

Number winsorized to upper bound: 3

Variable: T3 WSC

MAD: 1.8532499999999998

Median: 1.75

Lowerbound: -2.8831249999999997

Upperbound: 6.383125

no winsorization needed!

(3) Model of negative affect using earlier measurements (aka “lags”) as covariates:

Variable: T1 MIFU

MAD: 0.7413

Median: 3.7

Lowerbound: 1.8467500000000003

Upperbound: 5.55325

Winsorization done

new old higher/lower_bound

2 2.0 1.5 lower

44 2.0 1.6 lower

51 2.0 1.0 lower

62 2.0 1.2 lower

83 2.0 1.2 lower

92 2.0 1.7 lower

93 2.0 1.0 lower

107 2.0 1.2 lower

Number winsorized to lower bound: 8

Number winsorized to upper bound: 0

Variable: T1 ER

MAD: 0.6589333333333336

Median: 2.2222222222222223

Lowerbound: 0.5748888888888883

Upperbound: 3.8695555555555563

Winsorization done

new old higher/lower_bound

2 3.833333 3.888889 upper

20 3.833333 3.944444 upper

35 3.833333 4.000000 upper

38 3.833333 4.055556 upper

48 3.833333 4.388889 upper

51 3.833333 4.500000 upper

59 3.833333 4.333333 upper

60 3.833333 4.555556 upper

87 3.833333 4.000000 upper

89 3.833333 4.055556 upper

92 3.833333 5.000000 upper

93 3.833333 4.055556 upper

103 3.833333 4.222222 upper

107 3.833333 4.111111 upper

Number winsorized to lower bound: 0

Number winsorized to upper bound: 14

Variable: T2 ER

MAD: 0.6589333333333333

Median: 2.111111111111111

Lowerbound: 0.46377777777777807

Upperbound: 3.7584444444444443

Winsorization done

new old higher/lower_bound

13 3.722222 3.777778 upper

33 3.722222 3.777778 upper

48 3.722222 3.833333 upper

60 3.722222 4.333333 upper

87 3.722222 4.722222 upper

92 3.722222 5.000000 upper

93 3.722222 4.722222 upper

103 3.722222 4.111111 upper

107 3.722222 4.166667 upper

Number winsorized to lower bound: 0

Number winsorized to upper bound: 9

Variable: T1 NA

MAD: 0.8721176470588233

Median: 1.8823529411764706

Lowerbound: -0.2979411764705877

Upperbound: 4.062647058823529

Winsorization done

new old higher/lower_bound

2 4.058824 4.294118 upper

8 4.058824 4.411765 upper

48 4.058824 4.352941 upper

59 4.058824 4.823529 upper

62 4.058824 4.176471 upper

87 4.058824 4.588235 upper

92 4.058824 4.941176 upper

93 4.058824 4.882353 upper

Number winsorized to lower bound: 0

Number winsorized to upper bound: 8

Variable: T2 NA

MAD: 0.7849058823529413

Median: 1.7058823529411764

Lowerbound: -0.25638235294117684

Upperbound: 3.6681470588235294

Winsorization done

new old higher/lower_bound

0 3.647059 5.000000 upper

13 3.647059 3.941176 upper

22 3.647059 3.882353 upper

35 3.647059 3.705882 upper

48 3.647059 4.411765 upper

59 3.647059 4.058824 upper

60 3.647059 3.882353 upper

62 3.647059 5.000000 upper

83 3.647059 4.294118 upper

87 3.647059 4.764706 upper

92 3.647059 5.000000 upper

93 3.647059 5.000000 upper

103 3.647059 4.235294 upper

107 3.647059 4.823529 upper

123 3.647059 3.705882 upper

135 3.647059 4.176471 upper

144 3.647059 3.882353 upper

151 3.647059 3.764706 upper

158 3.647059 3.764706 upper

Number winsorized to lower bound: 0

Number winsorized to upper bound: 19

Variable: T3 NA

MAD: 0.9593294117647058

Median: 1.8823529411764706

Lowerbound: -0.5159705882352941

Upperbound: 4.280676470588235

Winsorization done

new old higher/lower_bound

38 4.235294 4.705882 upper

48 4.235294 4.411765 upper

51 4.235294 4.529412 upper

60 4.235294 4.529412 upper

62 4.235294 4.882353 upper

87 4.235294 4.705882 upper

92 4.235294 5.000000 upper

93 4.235294 4.588235 upper

103 4.235294 4.647059 upper

107 4.235294 4.294118 upper

Number winsorized to lower bound: 0

Number winsorized to upper bound: 10

# Appendix B

Details on the detection and treatment of univariate outliers in the second sensitivity analysis.

Variable: T1 MIFU

MAD: 0.7509417657492126

Median: 3.706503281902882

Lowerbound: 1.8291488675298506

Upperbound: 5.583857696275913

Winsorization done

new old higher/lower_bound

72 2.0 1.2 lower

107 2.0 1.2 lower

119 2.0 1.5 lower

126 2.0 1.0 lower

129 2.0 1.4 lower

130 2.0 1.2 lower

229 2.0 1.7 lower

231 2.0 1.6 lower

247 2.0 1.0 lower

Number winsorized to lower bound: 9

Number winsorized to upper bound: 0

Variable: T1 ER

MAD: 0.6589333333333333

Median: 2.2777777777777777

Lowerbound: 0.6304444444444446

Upperbound: 3.9251111111111108

Winsorization done

new old higher/lower_bound

6 3.888889 4.222222 upper

22 3.888889 4.000000 upper

44 3.888889 3.944444 upper

87 3.888889 4.055556 upper

96 3.888889 4.555556 upper

107 3.888889 4.111111 upper

122 3.888889 4.333333 upper

126 3.888889 4.055556 upper

142 3.888889 4.055556 upper

145 3.888889 4.000000 upper

163 3.888889 4.388889 upper

229 3.888889 5.000000 upper

247 3.888889 4.500000 upper

Number winsorized to lower bound: 0

Number winsorized to upper bound: 13

Variable: T2 ER

MAD: 0.7001166666666666

Median: 2.1666666666666665

Lowerbound: 0.41637499999999994

Upperbound: 3.916958333333333

Winsorization done

new old higher/lower_bound

6 3.833333 4.111111 upper

22 3.833333 4.722222 upper

96 3.833333 4.333333 upper

107 3.833333 4.166667 upper

126 3.833333 4.722222 upper

229 3.833333 5.000000 upper

Number winsorized to lower bound: 0

Number winsorized to upper bound: 6

Variable: T1 WSC

MAD: 2.2239

Median: 2.3333333333333335

Lowerbound: -3.2264166666666667

Upperbound: 7.893083333333333

no winsorization needed!

Variable: T2 WSC

MAD: 1.8532499999999998

Median: 1.6666666666666667

Lowerbound: -2.9664583333333328

Upperbound: 6.299791666666667

no winsorization needed!

Variable: T3 WSC

MAD: 2.10035

Median: 1.9166666666666667

Lowerbound: -3.3342083333333337

Upperbound: 7.167541666666668

no winsorization needed!

Variable: T1 NA

MAD: 0.784905882352941

Median: 1.8823529411764706

Lowerbound: -0.07991176470588202

Upperbound: 3.844617647058823

Winsorization done

new old higher/lower_bound

6 3.764706 3.941176 upper

7 3.764706 3.882353 upper

16 3.764706 3.941176 upper

22 3.764706 4.588235 upper

54 3.764706 4.180766 upper

72 3.764706 4.176471 upper

107 3.764706 4.058824 upper

119 3.764706 4.294118 upper

122 3.764706 4.823529 upper

125 3.764706 3.875000 upper

126 3.764706 4.882353 upper

143 3.764706 4.411765 upper

163 3.764706 4.352941 upper

228 3.764706 4.411765 upper

229 3.764706 4.941176 upper

240 3.764706 3.941176 upper

Number winsorized to lower bound: 0

Number winsorized to upper bound: 16

Variable: T2 NA

MAD: 0.8721176470588233

Median: 1.8235294117647058

Lowerbound: -0.35676470588235243

Upperbound: 4.003823529411764

Winsorization done

new old higher/lower_bound

6 3.941176 4.235294 upper

22 3.941176 4.764706 upper

54 3.941176 4.941176 upper

62 3.941176 4.062500 upper

72 3.941176 5.000000 upper

107 3.941176 4.823529 upper

122 3.941176 4.058824 upper

126 3.941176 5.000000 upper

130 3.941176 4.294118 upper

163 3.941176 4.411765 upper

196 3.941176 5.000000 upper

228 3.941176 4.470588 upper

229 3.941176 5.000000 upper

240 3.941176 4.176471 upper

Number winsorized to lower bound: 0

Number winsorized to upper bound: 14

Variable: T3 NA

MAD: 1.0901470588235294

Median: 2.0588235294117645

Lowerbound: -0.6665441176470588

Upperbound: 4.784191176470587

Winsorization done

new old higher/lower_bound

54 4.705882 5.000000 upper

72 4.705882 4.882353 upper

229 4.705882 5.000000 upper

Number winsorized to lower bound: 0

Number winsorized to upper bound: 3
